# Co-morbid risk factors and NSAID use among white and black Americans that predicts overall survival from diagnosed colon cancer

**DOI:** 10.1371/journal.pone.0239676

**Published:** 2020-10-07

**Authors:** Minoru Koi, Yoshiki Okita, Koki Takeda, Erika S. Koeppe, Elena M. Stoffel, Joseph A. Galanko, Amber N. McCoy, Temitope Keku, John M. Carethers

**Affiliations:** 1 Division of Gastroenterology and Hepatology, Department of Internal Medicine, University of Michigan, Ann Arbor, Michigan; 2 Division of Gastroenterology and Hepatology, Departments of Medicine & Nutrition, University of North Carolina at Chapel Hill, Chapel Hill, North Carolina; 3 Department of Human Genetics and Rogel Cancer Center, University of Michigan, Ann Arbor, Michigan; Howard University, UNITED STATES

## Abstract

Black Americans (BA) have higher incidence and higher mortality rates for colorectal cancers (CRC) as compared to White Americans (WA). While there are several identified risk factors associated with the development of CRC and evidence that high levels of adequate screening can reduce differences in incidence for CRC between BA and WA, there remains little data regarding patient co-morbid contributions towards survival once an individual has CRC. Here we set out to identify patient risk factors that influenced overall survival in a cohort of 293 BA and 348 WA with colon cancer. Amid our cohort, we found that patients’ age, tobacco usage, and pre-diagnosed medical conditions such as hypertension and diabetes were associated with shorter overall survival (OS) from colon cancer. We identified pre-diagnosed hypertension and diabetes among BA were responsible for one-third of the colon cancer mortality disparity compared with WA. We also identified long-term regular use of non-steroidal anti-inflammatory drugs (NSAIDs), including aspirin, was associated with shorter OS from colon cancer among WA >65 years of age, but not younger WA patients or any aged BA patients. Our results raise the importance of not only treating the colon cancer itself, but also taking into consideration co-morbid medical conditions and NSAID usage to enhance patient OS. Further evaluation regarding adequate treatment of co-morbidities and timing of NSAID continuance after cancer therapy will need to be studied.

## Introduction

In the United States, there is disparity for colorectal cancer (CRC) incidence and mortality between races, with Black Americans (BA) exhibiting higher rates as compared to White Americans (WA) [1–5]. Current evidence suggests that the contributions to this disparity is multi-factorial, intertwining socioeconomic issues that affect medical insurance coverage, access to preventive and regular medical care, use of tobacco and alcohol, and ingesting a high fiber, lower red meat diet that can affect the gut microbiome and cancer risk [2, 5–7]. Pathological and biological analyses show disparity in tumor stage contributing to earlier mortality, potential modification of the local tumor immunological environment, as well as unique somatic genetic mutations that appear isolated in BA CRC patients [1, 3, 5–9]. These contributions to BA CRC disparity are under investigation to understand their contribution towards the initial development of CRC, and to inform strategies to mitigate risk and the disparity [10, 11]. It has been shown that with intentional navigation-enacted CRC screening that the incidence and possibly mortality disparity can be erased [12].

However, there are few factors investigated regarding the contributions of conditions influencing the overall survival disparity in BA and WA once CRC is diagnosed. Certainly, some of the identified contributors for primary development of colorectal and disparity may also be factors in patient outcome after CRC is diagnosed. For instance, there is evidence that non-steroidal anti-inflammatory drugs (NSAIDs) including aspirin can aid primary prevention of CRC but also prevent secondary recurrence contributing to reduced mortality [13]. In addition, access to medical care once diagnosed, including treatment with surgery, chemotherapy and immunotherapy may not be equal based on socioeconomic conditions and can contribute to CRC disparity in outcome [3, 4, 10]. Because there are few published data regarding intrinsic contributors to patient overall survival, we explored non-socioeconomic factors that were focused on pre-existing medical conditions and use of NSAIDS and aspirin to assess if these contributed to the overall survival of WA and BA with CRC. Such information can inform caregivers on adjusting such factors that could influence overall survival in these CRC patients.

## Materials and methods

Data for colon cancer (CC) patients analyzed in this study was derived from a previously described study in which it was found that the use of non-steroidal anti-inflammatory drugs (NSAIDs) including aspirin was associated with a reduced incidence of CC [14]. All data was anonymized. The cohort consisted of 348 cases of WA and 293 cases of BA patients (Table 1). Patients were 40 to 84 years old, with their first diagnosis of invasive adenocarcinoma of the colon made between July 1, 1996 and June 30, 2000. Their medical histories and life-style factors such as tobacco use, alcohol use or NSAID use were collected through interviews [14]. Use of NSAIDs at least 5 years prior to diagnosis was recorded for each. Some users took multiple types of NSAID or took the same NSAID multiple times a day. We therefore calculated the aggregated years for all NSAID use (AYDU). NSAIDs used included 12 prescription and 9 over-the counter medications, including aspirin. Drug use was first assessed using the following two categories: regular use (1 time per day, ≥3 days per week), and occasional or rarely/no use (<3 day per week) for each of 21 drugs. For a drug regularly used, the exposure to the drug was expressed in years (EDY) calculated by a frequency of usage per day and duration of usage. For instance, the 1-year (1y) EDY could represent use of the drug 1 time per day for 1 year, 2 times per day for 6 months, or 3 times per day for 3.3 months. The sum of EDY for all drugs used by a patient (aggregated duration of NSAID use: ADYU) was calculated for each patient. Short-term regular (STR) use was defined when ADYU was 5y or less than 5y but longer than 0.008y (1 time per day for 3 days in a week). Long-term regular (LTR) use was defined when AYDU is longer than 5y. None use was defined when ADYU was less than 0.008y.

**Table 1:** Characteristics of White and Black American colon cancer patients.

		(N, %)	(N, %)	
Covariate		White Americans (N = 348)	Black Americans (N = 293)	*P*-value^a^
Gender	Male	194 (55.7)	138 (47.1)	0.032
	Female	154 (44.3)	155 (52.9)	
Age	≤65	160 (46.0)	169 (57.7)	0.003
	>65	188 (54.0)	124 (42.3)	
NSAID use	None/occasional	164 (47.1)	152 (51.9)	0.0048
	LTR	74 (21.3)	34 (11.6)	
	STR	110 (31.6)	107 (36.5)	
Smoking	Never	117 (33.6)	135 (46.1)	<0.0001
	Current	47 (13.5)	58 (19.8)	
	Former	181 (52.0)	97 (33.1)	
	Missing	3 (0.9)	3 (1.0)	
Alcohol^b^	No	204 (58.6)	221 (75.4)	<0.0001
	Yes	134 (38.5)	63 (21.5)	
	Missing	10 (2.9)	9 (3.1)	
Tumor site	Proximal	168 (48.3)	142 (48.5)	0.97
	Distal	157 (45.1)	133 (45.4)	
	Missing	23 (6.6)	18 (6.1)	
Tumor stage	Localized	123 (35.3)	96 (32.8)	0.562
	Regional	167 (48.0)	139 (47.4)	
	Distant	28 (8.1)	33 (11.3)	
	Missing	30 (8.6)	25 (8.5)	
Heart Problems	No	258 (74.1)	230 (78.5)	0.257
	Yes	86 (24.7)	61 (20.8)	
	Missing	4 (1.2)	2 (0.7)	
Hypertension	No	205 (58.9)	118 (40.3)	<0.0001
	Yes	140 (40.2)	173 (59.0)	
	Missing	3 (0.9)	2 (0.7)	
Diabetes	No	287 (82.5)	216 (73.7)	0.006
	Yes	58 (16.6)	75 (25.6)	
	Missing	3 (0.9)	2 (0.7)	
Arthritis	No	244 (70.1)	192 (65.5)	0.23
	Yes	101 (29.0)	99 (33.8)	
	Missing	3 (0.9)	2 (0.7)	

^a^ *P*-values were determined by chi-square test.

^b^ Any (yes) or no (NO) consumption of alcohols.

Abbreviations: NSAID, non-steroidal anti-inflammatory drug; LTR, long-term regular use; STR, short-term regular use.

Patient survival was followed for at least 10 years following diagnosis. The statistical associations between contributing factors were determined by the chi-square test and logistic regression test. Survival analysis was performed using the Kaplan-Meier method with log-rank test or Wilcoxon test and penalized Cox regression model (Cox Proportional Hazards Model, CPHM) with a Firth bias correction.

## Results

### Covariate factors associated with WA or BA Colon Cancer (CC) patients

**Fig 1:**
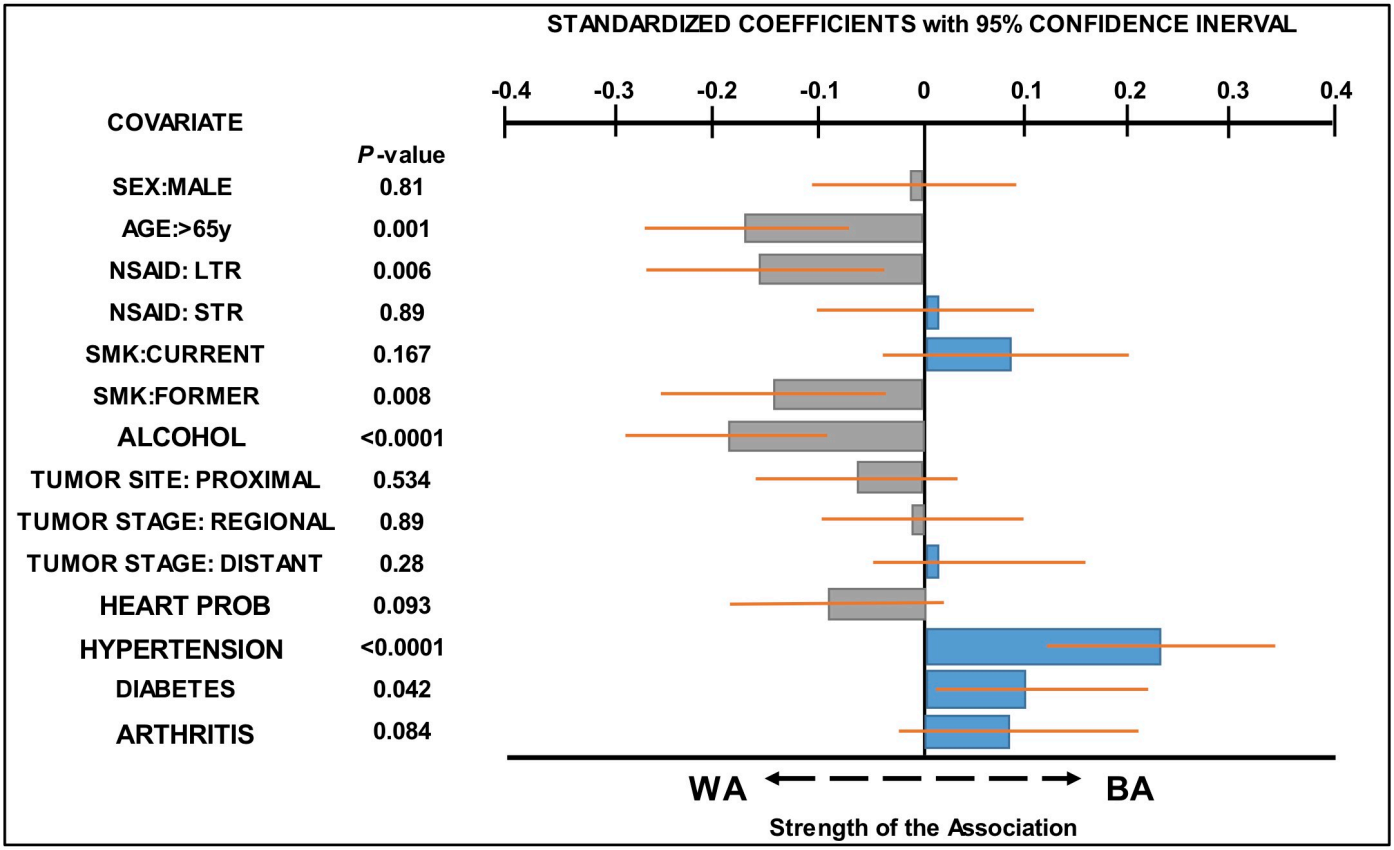
Covariates associated with BA and WA CC patients. The standardized coefficient for each covariate obtained from logistic regression model (*see* Table 2) was plotted to compare the strength of association of each covariate with BA CC or WA CC patients. The higher the value, the stronger the association with BA CC patients (blue bar) and the lower the value, the stronger the association with WA CC patients (gray bar). Orange lines represent a 95% confidence interval. P values represent the significance of the association between each covariate and BA or WA CC patients.

Covariate factors examined in this study, as well as the distribution of patients by race with characteristics defined by each covariate, are presented in Table 1. The association of patient’s race (WA or BA) CC with each covariate was determined using a multivariate logistic regression model (Fig 1 and S1 Table). The results showed that BA CC patients were younger than WA CC patients [odds ratio (OR): 0.54, 95%CI:0.38–0.77, p = 0.001] and were more likely to suffer from hypertension (HYP) (OR:2.31, 95%CI: 1.59–3.34, p<0.0001) or diabetes (OR: 1.57, 95%CI:1.02–2.41, p = 0.042), whereas more WA CC patients had quit smoking (SMK) (OR: 0.59, 95%CI:0.4–0.87, p = 0.008) than had BA CC patients. WA CC patients showed higher alcohol use (ALC) (OR: 0.46, 95%CI:0.31–0.69, p<0.0001) than their BA counterparts, and had used NSAIDs for a longer term (OD: 0.49, 95%CI:0.3–0.81, p = 0.006) (S1 Table). Fig 1 illustrates the relative strength of association between each covariate and WA or BA CC patients.

### Overall Survival (OS) rate of BA colon cancer patients is shorter than OS rate of WA colon cancer patients

**Fig 2:**
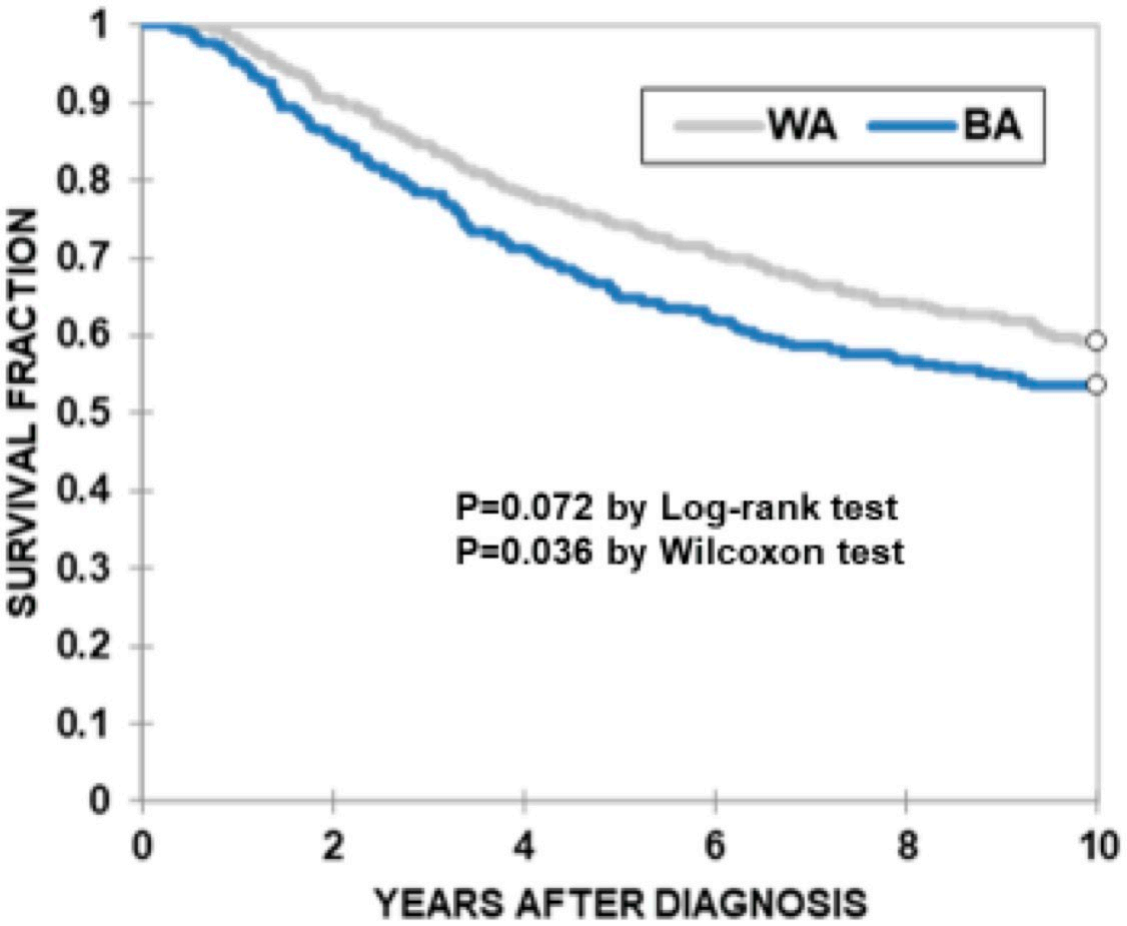
Kaplan-Meier curves for OS of CC patients. OS was compared between BA (*blue line*, n = 293) and WA (*gray line*, n = 348) CC patients. The X-axis represents years of OS after diagnosis. The Y-axis represents survival percentage. *P* values were determined by log-rank test (p = 0.072) or Wilcoxon test (p = 0.036).

We next determined whether there is a difference in the OS rate between WA and BA CC patients. The distribution of OS rate by the Kaplan-Meier method showed that BA CC patients exhibited a shorter OS rate than did WA CC patients (Fig 2: p = 0.072 by Log-rank test, p = 0.036 by Wilcoxon test). We then examined whether race was an independent factor associated with OS using Cox proportional hazards modeling (Table 2). In Model 1, the effect of race on OS was adjusted for age, sex, pre-diagnostic use of NSAIDs, medical conditions including diabetes, arthritis, heart problems (HRT-PRB) and HYP, tumor stage, tumor site, and life habits such as smoking (SMK) and alcohol (ALC) intake (Table 2). The results showed that BA CC patients had 29% higher risk for reduced OS than WA CC patients (Table 2, HR:1.29, 95%CI:1.00–1.67, p = 0.05). In Model 2 where covariates that did not have a significant effect (p>0.2) on OS in Model 1—sex, arthritis, HRT-PRB, and ALC—were not included, the association of BA CC patients to shorter OS rates reached a significant level (Table 2, HR:1.3, 95%CI:1.01–1.67, p = 0.039). Table 2 also shows that age, SMK, tumor site, tumor stage, HYP and diabetes are independent factors associated with OS rates in CC patients.

**Table 2:** Covariates associated with OS in CC patients.

**MODEL 1**				
Covariate		Hazard Ratio	95% Confidence Interval	*P*-value
Race	WA vs BA	1.29	1.00–1.67	0.05
Gender	Female vs Male	1.08	0.84–1.40	0.553
Age	≤65yrs vs >65yrs	1.57	1.21–2.03	0.001
Smoking	Never vs current	1.68	1.18–2.39	0.004
	Never vs former	1.44	1.07–1.92	0.015
Alcohol	No vs yes	0.96	0.72–1.28	0.794
NSAID	No vs LTR	1.25	0.90–1.72	0.179
	No vs STR	0.88	0.67–1.17	0.386
Tumor site	Proximal vs distal	1.32	1.03–1.71	0.032
Tumor stage	Localized vs regional	2.17	1.60–2.96	<0.0001
	Localized vs distant	7.42	5.02–10.97	<0.0001
Heart Problems	No vs yes	0.89	0.66–1.20	0.447
Hypertension	No vs yes	1.41	1.09–1.83	0.009
Diabetes	No vs yes	1.34	1.01–1.78	0.042
Arthritis	No vs yes	1.12	0.86–1.46	0.389
**MODEL 2**				
Race	WA vs BA	1.30	1.01–1.67	0.039
Age	≤65yrs vs >65yrs	1.59	1.23–2.04	<0.0001
Smoking	Never vs current	1.69	1.20–2.36	0.002
	Never vs former	1.49	1.13–1.97	0.005
NSAID	No vs LTR	1.26	0.92–1.71	0.147
	No vs STR	0.91	0.69–1.20	0.489
Tumor site	Proximal vs distal	1.33	1.03–1.71	0.027
Tumor stage	Localized vs regional	2.19	1.62–2.97	<0.0001
	Localized vs distant	7.48	5.06–11.05	<0.0001
Hypertension	No vs yes	1.39	1.08–1.78	0.01
Diabetes	No vs yes	1.38	1.04–1.83	0.026

Abbreviations: NSAID, non-steroidal anti-inflammatory drug; LTR, long-term regular use; STR, short-term regular use; WA, White American; BA, Black American.

Because we detected a disparity in OS between WA and BA CC patients, we next examined the contribution of each covariate to the observed disparity. Because HYP and diabetes were significantly associated with BA CC patients (Fig 1 and S1 Table) and both variables were independently associated with lower OS in our cohort (Table 2), we estimated the contribution of HYP or diabetes to the difference in OS between BA and WA CC patients. Comparing the multivariable hazard ratios (HR) for race adjusted for 5 variables without HYP (HR:1.37, 95%CI:1.07–1.76) and HRs for race adjusted by 6 variables including HYP (HR:1.3, 95%CI:1.01–1.67), 19.6% of the disparity in survival rate between white and black Americans was explained by HYP (Table 3). A similar comparison was made between HR for race adjusted using 5 variables, excluding diabetes (HR:1.34, 95%CI:1.05–1.72) and an HR for race adjusted using 6 variables including diabetes (HR:1.30, 95%CI:1.01–1.67). This indicated that there is 12% difference in survival rates between Black and White Americans that could be explained by diabetes (Table 3). These results indicate that approximately one third of the disparity between survival rates of Black and White American CC patients can be explained by hypertension and diabetes.

**Table 3:** Contribution of hypertension or diabetes to racial disparity in OS.

	Hazard Ratio, HR (95% CI)
HR for hypertension adjusted for 7 covariates^a^	1.39 (1.08–1.78)
HR for race adjusted for 5 covariates without hypertension	1.37 (1.07–1.76)
HR for race adjusted for 6 covariates with hypertension	1.30 (1.01–1.67)
**Proportion of racial disparity explained by hypertension** (%)	19.60%
HR for diabetes adjusted for 7 covariates^b^	1.38 (1.04–1.82)
HR for race adjusted for 5 covariates without diabetes	1.34 (1.05–1.72)
HR for race adjusted for 6 covariates with diabetes	1.30 (1.01–1.67)
**Proportion of racial disparity explained by diabetes** (%)	12%

^a^ Covariates include race, age, smoking, NSAID use, tumor site, tumor stage, and diabetes.

^b^ Covariates include race, age, smoking, NSAID use, tumor site, tumor stage, and hypertension.

### Older age and long-term regular use of NSAID use are associated with shorter OS in WA CC patients

**Fig 3:**
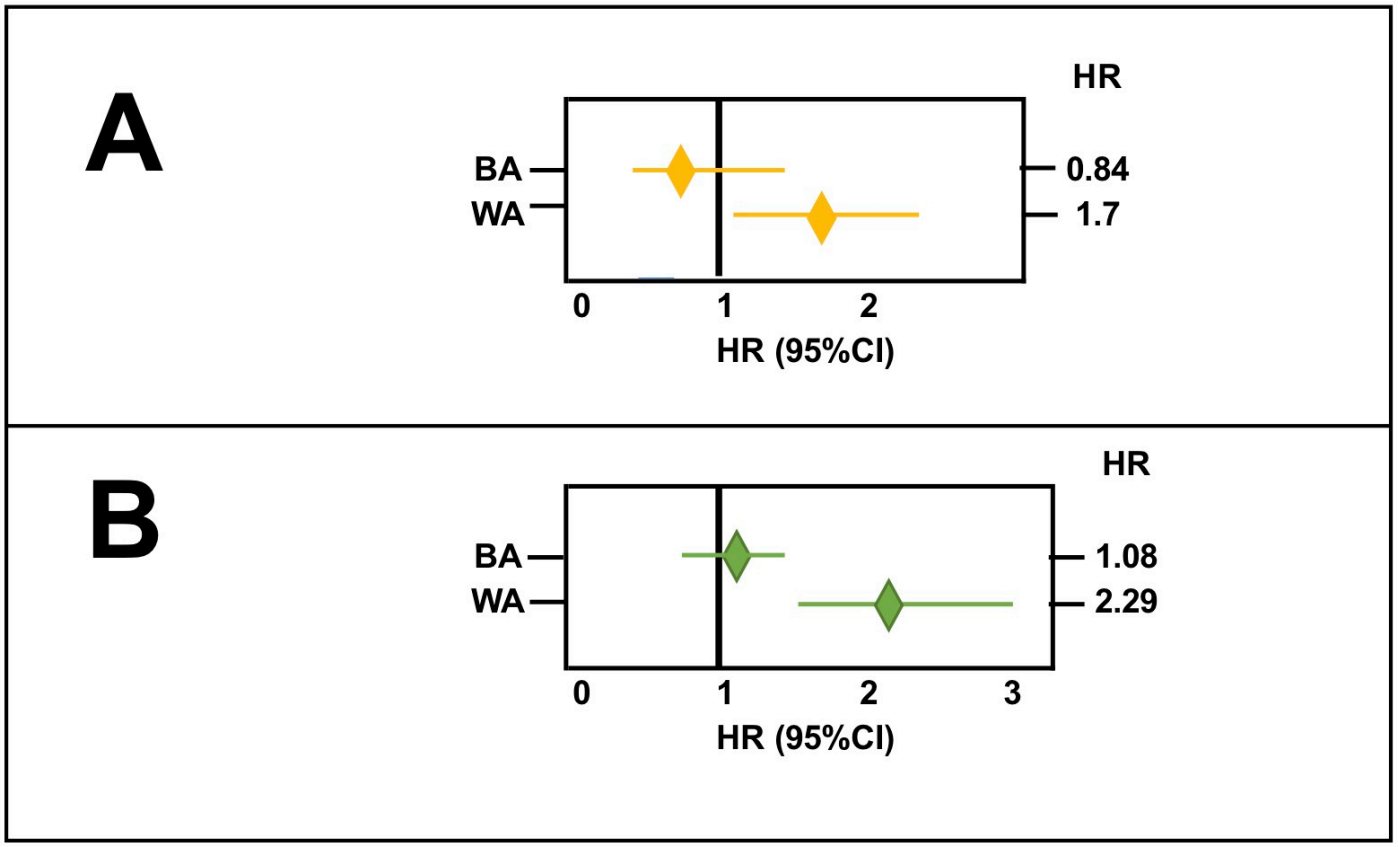
Estimated effects of LTR use of NSAIDs, and that of age, on OS in WA and BA CC patients. ***A***: Forest plot exhibiting the effect of LTR use of NSAIDs on OS in BA (HR:0.84, 95%CI:0.5–1.42) and WA (HR:1.7, 95%CI:1.15–2.51) CC patients. LTR use of NSAIDs had an opposite effect on BA, as opposed to WA CC patients. ***B***: Forest plot exhibits the effect of age on OS in BA (HR:1.08, 95%CI:0.77–1.51) and WA (HR:2.29, 95%CI: 1.58–3.32) CC patients. Age had a more critical effect on OS of WA than of BA CC patients. Each horizontal bar indicates 95% CI; the left end point shows the lowest value and the right end point the highest value of the confidence interval. The middle diamond shape indicates the value for hazard ratio.

In our CC cohort, survival rates related to LTR use of NSAIDs, but not STR use of NSAIDs, were found to interact with race (p_interaction_ = 0.038). Age was also found to interact with race (p_interaction_ = 0.004). The estimated HR for LTR use of NSAIDs in WA and BA indicated that LTR use of NSAIDs had an more effect on the OS rates for WA CC patients (HR:1.7, 95%CI:1.15–2.51), than for BA CC patients (HR:0.84, 95%CI:0.50–1.42) (Fig 3). Similarly, the estimated HR for age in WA and BA indicated that older age (>65y) had more effect on shorter OS in WA CC patients (HR:2.29, 95%CI:1.58–3.32) compared to BA CC patients (HR:1.08, 95%CI:0.77–1.51) (Fig 3). We therefore performed a subgroup analysis where the association between age and OS and the association between LTR use of NSAIDs and OS were examined in WA and BA CC patients separately. The results showed that older age (>65y) was associated with shorter OS in WA by univariate (HR:2.29, 95%CI:1.58–3.32) and multivariate analysis (HR:2.19, 95%CI:1.48–3.25) but not in BA CC patients (Table 4). LTR use of NSAIDs was also associated with shorter OS in WA by univariate (HR:1.69, 95%CI:1.15–2.51) and multivariate analysis (HR:1.55, 95%CI:1.02–2.37) but not in BA CC patients (Table 4).

**Table 4:** Older age and LTR use of NSAID are associated with shorter OS in WA CC patients.

		Total		WA CC Patients		BA CC Patients	
		N = 641		N = 348		N = 293	
		Hazard Ratio (95% CI)	*P* value	Hazard Ratio (95% CI)	*P* value	Hazard Ratio (95% CI)	*P* value
Univariate	Age (≤65yrs vs >65yrs	1.49 (1.17–1.89)	0.001	2.29 (1.58–3.32)	<0.0001	1.08 (0.77–1.51)	0.68
	NSAID (non vs LTR)	1.25 (0.93–1.69)	0.15	1.69 (1.15–2.51)	0.008	0.84 (0.50–1.42)	0.52
Multivariate^a^	Age (≤65yrs vs >65yrs	1.50 (1.17–1.94)	0.002	2.19 (1.48–3.25)	<0.0001	1.25 (0.87–1.81)	0.23
	NSAID (non vs LTR)	1.22 (0.88–1.68)	0.23	1.55 (1.02–2.37)	0.042	0.81 (0.47–1.42)	0.16

^a^ Age and NSAID use are adjusted for gender, heart problems, hypertension, diabetes, arthritis, smoking, alcohol intake, tumor site, and tumor stage.

Abbreviations: NSAID, non-steroidal anti-inflammatory drug; LTR, long-term regular use; WA, White American; BA, Black American; CI, confidence interval.

### WA CC elderly patients (>65 years of age) with pre-diagnostic LTR use of NSAIDs are at high risk for lower OS rates

**Fig 4:**
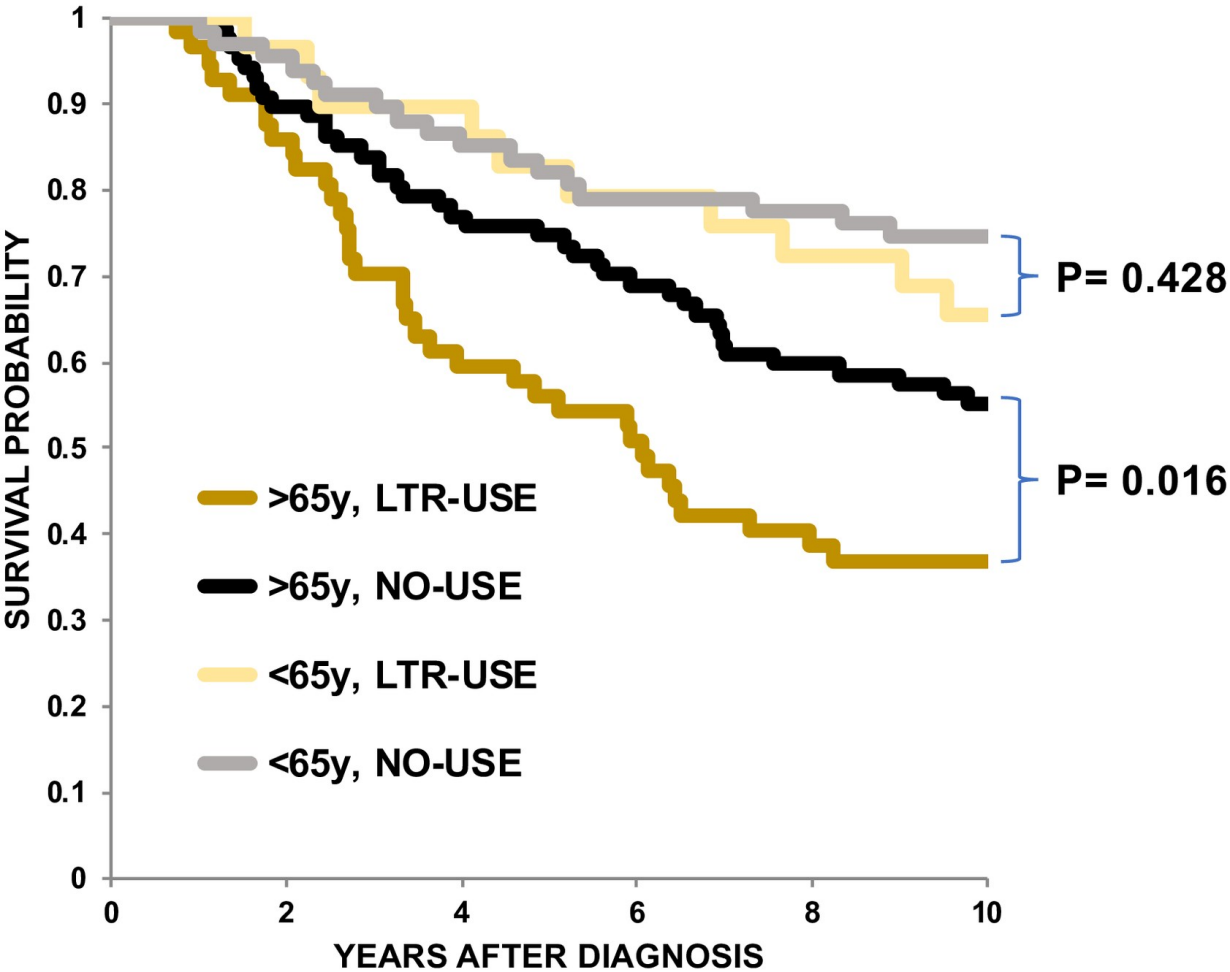
Kaplan-Meier curves for OS of WA CC patients. OS rates were compared among WA CC patients with LTR use of NSAIDs who were older than 65 years (*mustard line*, n = 57), with patients older than 65 with no (NO) use of NSAIDs (black line, n = 87), with patients 65 years or younger with LTR use of NSAIDs (yellow line, n = 29) and patients 65 years or younger with no (NO) of NSAIDs (*gray line*, n = 67). The X-axis represents years of OS after diagnosis. The Y-axis represents survival probability. *P* values were determined by log-rank test.

We found that older age (>65y) and LTR use of NSAIDs were associated with shorter OS in WA CC patients (Table 4). To determine whether the effect of NSAID use on OS was dependent on age, we divided the WA CC patients into 4 groups: >65y and LTR use (>65y/LTR), >65y and no use (>65y/none), ≤65y and LTR use (≤65y/LTR), and ≤65y and no use (≤65y/none). We detected a significant difference in the OS rates between >65y/LTR and >65y/no (p = 0.016) but not between ≤65y/LTR and ≤65y/no (p = 0.428) by log-rank test (Fig 4), indicating that LTR use of NSAIDs was associated with shorter OS if patients were older than 65y compared to patients younger than 65. These results indicate that the effect of LTR use of NSAIDs depends on age. We therefore examined the association between LTR use of NSAIDs in younger (≤65y) and in older (>65y) WA CC patients separately using Cox proportional hazard modeling. Table 5 shows that older WA CC patients (>65y) who were LTR but not STR users of NSAIDs exhibited shorter OS compared to WA CC patients who were not NSAID users by univariate (HR:1.76, 95%CI: 1.12–2.77, p = 0.015) and by multivariate CPHM (HR:1.82, 95%CI:1.12–2.97, p = 0.016).

**Table 5:** Older but not younger WA CC patients with LTR use of NSAID is associated with shorter OS.

		≤65 years		>65 years	
		N = 145	*P* value	N = 203	*P* value
		Hazard Ratio (95% CI)		Hazard Ratio (95% CI)	
Univariate	None vs LTR	1.37 (0.63–2.98)	0.44	1.76 (1.12–2.77)	0.015
	None vs STR	0.90 (0.42–1.93)	0.79	1.08 (0.67–1.75)	0.76
Multivariate^a^	None vs LTR	1.08 (0.42–2.74)	0.87	1.82 (1.12–2.97)	0.016
	None vs STR	0.62 (0.27–1.43)	0.26	0.92 (0.56–1.53)	0.75

^a^ NSAID use was adjusted for gender, heart problems, hypertension, diabetes, smoking, and tumor stage.

Abbreviations: NSAID, non-steroidal anti-inflammatory drug; LTR, long-term regular use; STR, short-tern regular use; CI, confidence interval.

## Discussion

In this study, we detected disparity in OS between BA and WA CC patients (Table 2 and Fig 2). To determine whether any factors we examined explained the racial disparity in OS, we first performed logistic regression modeling to identify covariates associated with BA or WA CC patients. The results showed that a higher percentage of patients in the BA CC population suffered from HYP and diabetes than did the WA CC population (Fig 1 and S1 Table). Furthermore, our results showed that HYP and diabetes were independent factors that were associated with shorter OS in all CC patients (Table 2). We therefore calculated the percent at which HYP and diabetes contributed to the racial disparity in OS between WA and BA CC patients. The results showed that HYP and diabetes explained 19.6% and 12% of disparities, respectively (Table 3), suggesting that there are additional factor(s) that explain the remaining two-thirds of disparity between WA and BA [1–7]. Although HYP or diabetes alone is not a main contributor to the racial disparity in OS for our cohort, our results show that HYP is an independent factor for shorter OS in CC (Table 2). Yang *et al* reported that HYP was significantly associated with shorter OS and lower recurrence-free survival in a large cohort of CC patients, suggesting that HYP may directly and/or indirectly promote recurrence after surgery of CC [15]. Diabetes is also an independent factor associated with shorter OS in our CC patients (Table 2). The association between pre-existing diabetes and shorter OS in colorectal cancer (CRC) patients has been shown in several studies [16, 17]. However, the effect of diabetes on cancer-specific survival (CSS) has not been clear. Some but not all studies demonstrate a significant association between shorter CSS and diabetes in CRC patients [17–19]. Thus, deaths of CRC patients with diabetes may stem from either complication from diabetes or the results of unidentified interactions between CRC and diabetes. Regarding smoking, in our CC cohort, current and former SMK were also independent factors associated with shorter OS (Table 2). In CRC patients, current and former smoking at the time of diagnosis has been associated with shorter OS compared to never smoking [20–22]. Current smoking, but not former smoking, was associated with CSS in one study [22], and neither current nor former smoking was associated with CSS in another study [21]. Overall, our results are compatible with the idea that smoking has a detrimental effect on survival of CRC patients after diagnosis.

One of the new findings of our study is that older age is a prognostic factor for shorter OS in WA but not in BA CC patients (Table 4). This difference could be due to the fact that the BA CC patients in our cohort were younger than the WA CC patients (Table 1). The impact of age on OS can be explained by increased deaths from other causes, such as comorbidity [23, 24] and/or undertreatment [25], rather than by tumor progression. However, our second finding raises the possibility that age may biologically interact with other factors such as LTR use of NSAID to worsen existing CC.

The second finding is that LTR but not STR use of NSAIDs is associated with shorter OS in WA but not BA CC patients (Table 4). A larger percentage of WA CC patients were LTR users of NSAIDs before diagnosis than were BA CC patients (Fig 1). Furthermore, the association between LTR use of NSAIDs and shorter OS in WA CC patients was age-dependent (Table 5 and Fig 4). These results raise two possibilities. First, the observed effect of LTR of NSAIDs on shorter OS may be confounded by cardiovascular comorbidities. In fact, HRT-PRB is associated with LTR use of NSAIDs (OR: 3.35, 95%CI: 0.56–1.86, p<0.0001, not shown); however, it is not associated with shorter OS in WA CC patients (HR:0.89 95%CI: 0.59–1.34) (Table 2). Alternatively, because most CC patients in our cohort must have carried undiagnosed CC when they were taking NSAIDs, the exposure of such tumors to NSAIDs for longer periods may increase tumor aggressiveness in elderly WA population. A recent randomized controlled trial (RCT), ASPirin in Reducing Events in the Elderly (ASPREE), showed that the regular use of low dose aspirin (LDA) was associated with increased colorectal cancer mortality in a healthy elderly population [26]. Another RCT trial, the Japanese Primary Prevention Project (JPPP), also failed to show any preventative effects of regular use of LDA but showed earlier cancer diagnoses in elderly participants who took LDA regularly [27]. Assuming that the elderly population may include more participants with undiagnosed cancers, the observed effects of LDA use in the ASPREE and JPPP cohorts could be attributed to enhanced progression of undiagnosed cancers exposed to LDA in elderly population [28]. In support of this view, McNeil et al recently showed that the percentage of metastatic cancers and mortality rates of patients with stage III and IV cancers increased in patients exposed to LDA compared to patients not exposed to LDA in the ASPREE cohort [29]. They speculated that the suppressive effect of LDA use on anti-tumor immunity might be augmented in elderly patients, whose immunity is already compromised, leading to increased metastasis [29–31]. They also proposed that genetic and/or epigenetic alterations in cancers in elderly patients might be different than in cancers from younger patients, which might render tumor cells from elderly patients more resistant to the anti-tumor effects of LDA [29].

In our cohort, we also detected a higher percentage of stage IV CC in the high-risk group of WA who were older than 65y and LTD users (12.9%: 7/54 cases) compared to the low-risk group of WA who were older than 65y and NSAID non-users (7.14%: 2/28 cases); however, the difference was not significant (p = 0.4). This could be due to the small sample size. In the ASPREE cohort, the adverse effect of LDA was limited to white Australians but not US participants, suggesting that racial or ethnic factors may have influenced the observed effects. In our cohort, we detected racial differences in OS between WA elderly LTR users and the similar elderly BA group. To explain our observations, we propose another possibility for the differential effects of regular use of aspirin and NSAIDs in elderly versus young, and WA versus BA populations. Zhang et al showed that lack of platelet-derived growth factor B (PDGFB) in platelets impaired the integrity of tumor vessels, resulting in enhanced hypoxia and epithelial-to-mesenchymal transitions in primary sites, increased circulating tumor cells and distant metastasis [32]. Lack of, or reduced levels of PDGFB in primary CC sites, may be also induced by regular use of aspirin/NSAIDs that block platelet activation. This may weaken the barrier function of tumor vessels, resulting in increased intravasation of primary tumor cells. Because the endothelial barrier function is weakened due to aging [33], the chance of extravasation of disseminated tumor cells may be higher in elderly patients as compared to younger patients. It has been reported that BA have higher counts and reactivity of platelets compared to WA [34, 35]. Thus, the inhibitory effects of aspirin/NSAIDs on platelet activation could be less in BA than in WA patients, resulting in a less porous tumor microenvironment and less intravasation of tumor cells from primary sites in BA. To explore these possibilities, further molecular and immunological analyses of bio-samples from elderly patients exposed to aspirin and/or NSAIDs, as well as creation of an animal model of aspirin/NSAID induced metastasis, are needed.

Overall, our results showed that there is a disparity in OS between WA and BA CC patients. Our results suggest that CC patients, regardless of race, who were former or current smokers, or who had heart problems, hypertension, or diabetes before diagnosis, had a high risk of having shorter OS. In WA but not BA CC patients, older age and LTR use of NSAIDs including aspirin were risk factors for shorter OS, and elderly WA CC patients who were LTR user of NSAIDs were at especially high risk for shorter OS. Our results raise the importance of not only treating cancer itself, but also of treating patients holistically, taking into account individual characteristics. Further study will be needed to confirm our findings.

## Supporting information

S1 Table Association of each covariate with BA CC patients compared to WA CC patients. (PDF)

## Abbreviations

ALC: alcohol
AYDU: aggregated years of drug use
BA: Black American
CC: colon cancer
CPHM: Cox proportional hazard model
CRC: colorectal cancer
CSS: cancer specific survival
HR: hazard ratio
HRT-PRB: heart problem
HYP: hypertension
LDA: low dose aspirin
LTR: long-term regular use
NO: non-user
NSAID: non-steroidal anti-inflammatory drug
OR: odds ratio
OS: overall survival
RCT: randomized controlled trial
SMK: smoker
STR: short-term regular user
WA: White American
