# Supplementary material for: Co-morbid risk factors and NSAID use among white and black Americans that predicts overall survival from diagnosed colon cancer
Source: PLoS One. 2020 Oct 7;15(10):e0239676. doi: 10.1371/journal.pone.0239676 (PMC7540856; doi:10.1371/journal.pone.0239676)
Supplement: S1 Table — (PDF) [file pone.0239676.s001.pdf]

Supplementary Data
for

Co-Morbid Risk Factors and NSAID Use Among White and Black Americans That Predicts Overall Survival from Diagnosed Colon Cancer

[1*]Minoru Koi, [1*]Yoshiki Okita, [1]Koki Takeda, [1]Erika Koeppe, [1]Elena M. Stoffel, [2]Joseph A Galanko, [2]Nikki McCoy, [2]Temitope Keku, [1,3#]John M Carethers

[1]Division of Gastroenterology and Hepatology, Department of Internal Medicine, University of Michigan, Ann Arbor, Michigan
[2]Division of Gastroenterology and Hepatology, Departments of Medicine & Nutrition, University of North Carolina at Chapel Hill, Chapel Hill, North Carolina
[3]Department of Human Genetics and Rogel Cancer Center, University of Michigan, Ann Arbor, Michigan

Supplementary Table 1 (S1 Table)

Association of Each Covariate with BA CC Patients Compared to WA CC Patients

Covariates		Odds Ratio	95%CI	*P* value
SEX				
	Male	0.96	0.67-1.38	0.81
AGE				
	>65y	0.54	0.38-0.77	0.001
NSAID USE				
	LTR	0.49	0.3-0.81	0.006
	STR	1.03	0.7-1.51	0.89
SMOKING				
	Current	1.44	0.86-2.42	0.17
	Former	0.59	0.4-3.37	0.008
ALCHOL				
	Yes	0.46	0.31-0.69	<0.0001
TUMOR SITE				
	Distal	0.81	0.57-1.15	0.24
TUMOR STAGE				
	Regional	0.97	0.66-1.43	0.89
	Distant	1.41	0.75-2.64	0.28
HEART PROBLEMS				
	Yes	0.68	0.44-1.07	0.09
HYPERTENSION				
	Yes	2.31	1.59-3.34	<0.0001
DIABETES				
	Yes	1.57	1.02-2.41	0.042
ARTHRITIS				
	Yes	1.41	0.96-2.08	0.084

abbreviation; LTR: long term regular use, STR: short term regular use
